# World Health Organization Versus Diabetes in Pregnancy Study Group India Approaches in the Screening of Gestational Diabetes Among Pregnant Women With Risk Factors: A Study Among Rural Population of Telangana, South India

**DOI:** 10.7759/cureus.29799

**Published:** 2022-09-30

**Authors:** Ayesha Jabeen, Amtul Rahman Amberina, Ahlad Sreedhrala, Dinesh Eshwar Mummareddi, Gulam Saidunnisa Begum

**Affiliations:** 1 Biochemistry, Mahavir Institute of Medical Sciences, Hyderabad, IND; 2 Biochemistry, College of Medicine and Health Sciences, Sohar, OMN

**Keywords:** oral glucose tolerance test, pregnant women, rural population, diagnose, diabetes in pregnancy study group india (dipsi), world health organization (who), gestational diabetes mellitus

## Abstract

Background

Gestational diabetes mellitus (GDM) represents a pathological condition wherein pregnant women (PW) suffer from glycemic dysregulation, which predisposes them to an increased risk of developing complications related to pregnancy and childbirth. The most commonly used guidelines to screen for GDM include those provided by the World Health Organization (WHO), the American Congress of Obstetricians and Gynecologists, the Canadian Diabetes Association, and the International Association of Diabetes and Pregnancy Study Group. The Diabetes in Pregnancy Study Group India (DIPSI) guidelines are national-level recommendations to screen for GDM in India. This study aimed to compare the efficacy of DIPSI criteria versus the WHO guidelines in screening for GDM among the rural population of Telangana, South India

Methods

A total of 300 PW aged 19-35 years with a gestational age of 24-28 weeks attending the antenatal clinic attached to Mahavir Institute of Medical Sciences (MIMS), Vikarabad, Telangana, India were included in the study. The study was approved by the Institutional Ethics Committee of MIMS, and informed consent was obtained from all the participants. Of the 300 subjects included, 75 PW were categorized as at-risk for GDM based on risk factors and were included for further analysis. The data relating to body mass index (BMI), oral glucose tolerance test, and the diagnosis of GDM based on DIPSI and the WHO criteria were collected.

Results

Out of the 75 PW included in the study, an overall GDM prevalence of 32% was noted among which 20 (26.7%) were diagnosed using the WHO criteria, 12 (16%) by DIPSI criteria, and the remaining 73.3% were non-GDM women. The mean gestational age and BMI among non-GDM and GDM patients were 24.74±4.15 weeks, 22.24±3.60 kg/m^2^, and 25.70±4.40, 24.48±3.37 kg/m^2^ (p<0.01), respectively. The activities of glucose at the second hour after a GTT among non-GDM and GDM cases were 113.70±20.4 mg/dL and 128.04±18.6 mg/dL (p=0.004), respectively.

Conclusion

DIPSI criteria could identify fewer numbers of GDM women as compared to the WHO criteria. Although the DIPSI criteria are convenient and prescribe less number of interventions, they could possibly miss many cases of GDM. Moreover, PW who remain undiagnosed could, in the future, be at risk of developing diabetes. Based on the study results and because risks should outweigh the benefits, we propose that DIPSI cannot be implemented as a single criterion to screen for GDM among PW in Indian settings.

## Introduction

Dysfunctional glucose metabolism results in gestational diabetes mellitus (GDM) among pregnant women (PW) and affects 3-10% of pregnancies. GDM presents as glucose intolerance of variable degrees with onset or first recognition during pregnancy accounting for 90% of cases of diabetes mellitus (DM) in pregnancy [1]. Mothers diagnosed with GDM and the children born to them are predisposed to DM in the future [2]. GDM is usually diagnosed during the third trimester of pregnancy and clinically resembles type 2 DM (T2DM), wherein patients show signs of insulin resistance with beta-cell dysfunction [3]. 

As per the International Diabetes Federation (IDF), Diabetes Atlas 2015, one in every seven births is affected by GDM. India is labeled as the diabetes capital of the world with more than 69.2 million cases including four million women with GDM alone [4,5]. The prevalence of GDM is reported to vary widely (3.8-21%) depending on the geographical location and the diagnostic criteria used in different parts of India [6]. It is estimated that there are 76 million women between the age of 20 and 39 years who have diabetes/pre-diabetes and are therefore at risk of suffering a pregnancy complicated by diabetes. Moreover, an increasing number of women who are at risk of developing GDM may be misdiagnosed during pregnancy because of inappropriate screening and lack of awareness [7].

In 1999, the World Health Organization (WHO) introduced criteria for diagnosis of GDM based on a 2-hour venous plasma glucose (VPG) cutoff value of 140 mg/dL (7.8 mmol/L), after the administration of 75 g of glucose orally [8]. In 2010, based on the findings of the Hyperglycemia and Adverse Pregnancy Outcomes (HAPO) study group and earlier observational studies, the International Association of Diabetes and Pregnancy Study Group (IADPSG) proposed more stringent diagnostic thresholds to diagnose GDM. It was recommended that a fasting plasma glucose level ≥5.1 mmol/L and/or 1-hour plasma glucose level ≥10.0 mmol/L and/or 2-hour plasma glucose level ≥8.5 mmol/L be used to diagnose GDM. These recommendations were adopted by the American Diabetes Association in 2010, the International Federation of Gynecology and Obstetrics in 2015, and recommended by the WHO [9,10].

The Diabetes in Pregnancy Study Group India (DIPSI) recommends testing 2-hour VPG after a 75 g oral glucose load, known as the oral glucose tolerance test (OGTT). According to the DIPSI criteria, a 2-h VPG ≥ 140 mg/dL (7.8 mmol/L) under non-fasting conditions is to be used to diagnose GDM. The major difference between the WHO recommended criteria with that of the DIPSI criteria is that in the former method, the PW are required to be in a fasting state. However, the DIPSI criteria are convenient and an OGTT can be performed both in fasting/non-fasting situations irrespective of the last meal timing [11]. The DIPSI criteria have gained increased acceptance in the Indian setup because of its simpler methods to diagnose GDM as compared to the two-step, and three-step procedures advocated by the Canadian Diabetes Association/IADPSG, and the American Congress of Obstetricians and Gynecologists, respectively.

The DIPSI criteria are cost-effective, and a single-step screening and diagnostic tool that does not need fasting, unlike the WHO guidelines [12]. All the PW who walk into the clinic can be screened, and this appears to best suit rural populations who have restricted access to quality antenatal health care. However, it remains unclear if using a single criterion like the DIPSI is appropriate for the diagnosis of GDM. Therefore, this study was carried out to compare the utility of the DIPSI criteria with the WHO guidelines in the screening for GDM among at-risk PW in the rural population of Telangana.

## Materials and methods

This cross-sectional study was carried out between June and August 2021. A total of 300 PW of 24-28 weeks of gestational age attending the antenatal outpatient department of Mahavir Institute of Medical Sciences (MIMS), Vikarabad, Telangana, India, were enrolled in the study. The sample size was estimated using online software (https://www.calculator.net/sample-size-calculator.html). A minimum of 73 participants were required when the prevalence rate was taken as 5%, with an error rate of 5% and at a confidence interval of 95% [n=Z^2^Xp̂(1-p̂)/ε^2^]. Of the 300 PW, 75 were identified as a risk group who may be predisposed to GDM and were included in the study for further analysis. The study was approved by the Institutional Ethics Committee of MIMS (MIMS/IEC/2018/026). All the participants of the study have been duly explained the process and informed consent was taken.

Study inclusion and exclusion criteria

PW aged between 19 and 35 years, who were within 24-28 weeks of gestational age and singleton pregnancy, have been preliminarily selected as study subjects. All the participants were then assessed for one or more of the following risk factors for GDM such as the history of GDM in previous pregnancies, BMI ≥25 kg/m^2^, first-degree relative with T2DM, polycystic ovarian syndrome, precious pregnancy, women with excessive weight gain during pregnancy, previous macrosomia baby (baby's weight ≥4 kg), and previous history of recurrent miscarriages, and congenital anomalies. PW with these risk factors for GDM were identified and included in the study for further analysis. All women who were aged <19 years and ˃35 years, and those who have been diagnosed with T2DM, hypertension, hypothyroidism, acromegaly, and women who were prescribed medications like steroids, anti-hypertensives, antidiabetics, antipsychotics, and diuretics, among others, were excluded from the study.

Data collection procedure 

Detailed current and previous obstetric history along with the family history of DM in parents and siblings were collected. History of smoking and alcohol intake was also recorded on their first visit (see Appendices). Further, other relevant history was recorded (see Appendices). Body mass index (BMI) was calculated (kg/m²) in all the study subjects. An OGTT was performed as per the DIPSI criteria wherein each study participant was given 75 g of glucose dissolved in 300 mL of water. After a 2-hour period, blood samples were collected and analyzed by the glucose oxidase peroxidase method using an endpoint colorimetric principle in a semi-automated analyzer. 

Three days later, the same group of subjects were asked to visit the lab for an OGTT and were instructed to ensure 12 hours of overnight fasting as per the latest WHO guidelines. On the day of the test, fasting venous blood samples were collected from each participant. After an OGTT, the patients were advised to remain on the hospital premises during the waiting period of 2 hours without any active exercise in both methods. Later, blood samples were collected from each participant at 1-hour and 2-hour intervals, and the activities of glucose were estimated. The diagnostic criterion for GDM based on different guidelines is shown in Table 1.

**Table 1:** Methods to diagnose gestational diabetes using various guidelines/criteria OGTT: oral glucose tolerance test; DIPSI: Diabetes in Pregnancy Study Group India; WHO: World Health Organization; IADPSG: International Association of Diabetes and Pregnancy Study Group. Conversion: 1 mmol/L=18 mg/dL.

Guideline/criteria	Fasting blood glucose (mmol/L)	Glucose challenge/OGTT	1-hour blood glucose (mmol/L)	2-hour blood glucose (mmol/L)	3-hour blood glucose (mmol/L)
DIPSI	Not required	75 g	Not required	≥7.8	Not required
WHO, 2013	≥5.1	75 g	≥10.0	≥8.5	Not required
WHO, 1993	≥7.0	75 g	Not required	≥7.8	Not required
American Congress of Obstetricians and Gynecologists	≥5.3	100 g	≥10.0	≥8.6	≥7.8
Canadian Diabetes Association	≥5.3	75 g	≥10.6	≥8.9	Not required
IADPSG	≥5.1	75 g	≥10.0	≥8.5	Not required

Statistical analysis 

The collected data were entered into a Microsoft Office 2019 Excel sheet (Microsoft® Corp., Redmond, WA), and were used to prepare tables and calculate the mean and percentages. Data were analyzed using SPSS software version 20 (IBM Corp., Armonk, NY) and a Fisher's exact test and chi-square test were applied to find out the significance. A p-value of <0.05 was considered to be significant.

## Results

Of the total 75 PW with risk factors for the development of GDM recruited for the study, 24 (32%) were identified as suffering from GDM, and 51 (68%) were non-GDM subjects. The mean gestational age and BMI among non-GDM and GDM patients were 24.74±4.15 weeks, 22.24±3.60 kg/m^2^, and 25.70±4.40 weeks, 24.48±3.37 kg/m^2^ (p=0.01), respectively. The mean OGTT at 2 hours was significantly higher (p=0.004) among GDM patients (128.04±18.6 mg/dL) as compared to the non-GDM subjects (113.70±20.4 mg/dL) as shown in Table 2.

**Table 2:** Gestational age, BMI, and OGTT results of the study subjects GDM: gestational diabetes mellitus; BMI: body mass index; OGTT: oral glucose tolerance test. *Statistically significant.

Variable	Non-GDM (n=51)	GDM (n=24)	p-Value
Gestational age (weeks)	24.74±4.15	25.70±4.40	0.7
BMI (kg/m^2^)	22.24±3.60	24.48±3.37	0.01*
OGTT at 2 hours (mg/dL)	113.70±20.4	128.04±18.6	0.004*

**Figure 1:**
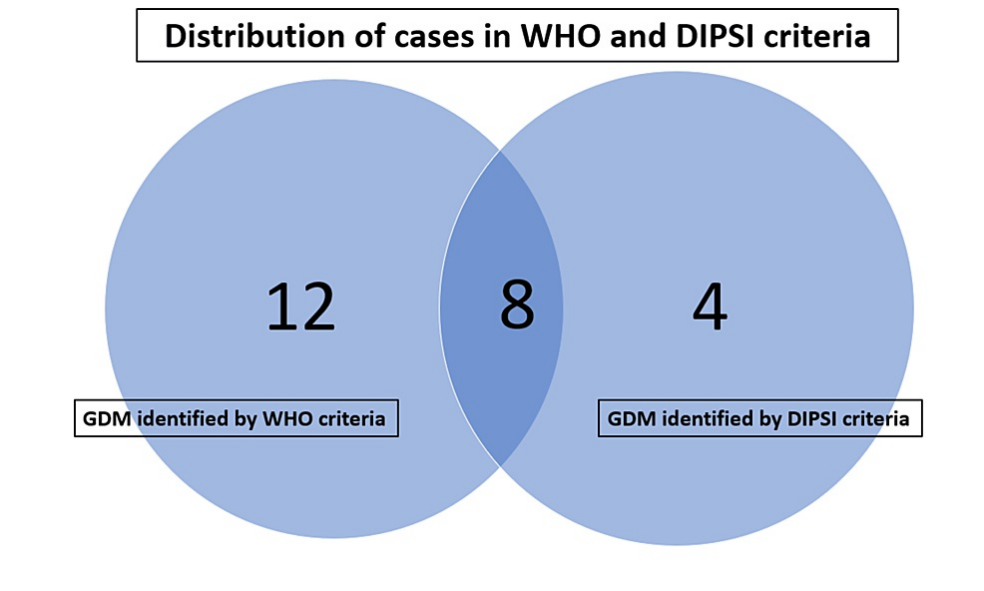
Venn diagram showing the detailed breakup of patients diagnosed with GDM based on the WHO and DIPSI criteria GDM: gestational diabetes mellitus; DIPSI: Diabetes in Pregnancy Study Group India; WHO: World Health Organization; IADPSG: International Association of Diabetes and Pregnancy Study Group.

Among the 24 PW who were diagnosed as suffering from GDM, 8 PW returned positive for GDM both by the WHO and DIPSI criteria. However, 12 were identified by WHO, and four were identified by DIPSI criteria alone as shown in Figure 1.

In our study we found that 19 cases were identified as GDM using the fasting blood sugar (FBS) and only two cases were identified by the OGTT at the second^ ^hour. No cases were identified by using the first-hour blood glucose value using the WHO criteria. Conversely, the DIPSI criteria identified 12 cases of GDM based on the second-hour blood glucose values after OGTT. The prevalence of GDM in the risk population was 26.7% using the WHO approach and 16% using the DIPSI criteria as shown in Table 3.

**Table 3:** Prevalence of GDM observed among the study participants GDM: gestational diabetes mellitus; DIPSI: Diabetes in Pregnancy Study Group India; WHO: World Health Organization.

Guidelines/criteria applied to identify GDM	Number of cases identified	Prevalence (%)
WHO	20	26.7
DIPSI	12	16

## Discussion

PW diagnosed with GDM are at an increased risk of developing T2DM. Moreover, the children born to such mothers are predisposed to T2DM. PW with GDM require intensive monitoring during pregnancy to offset the potential complications. Therefore, it is extremely important to carry out an accurate diagnosis of GDM. Earlier, a two-step approach was followed for the diagnosis of GDM, which used initial screening with the 50 g glucose challenge test (GCT), succeeded by an OGTT in patients with abnormal GCT results. The HAPO study and the subsequent IADPSG criteria recommended the use of OGTT for the diagnosis of GDM. However, this proved to be an impractical exercise, especially in developing and financially constrained countries. Hence, the DIPSI suggested a 75 g OGTT that does not require a fasting sample, which was an economically feasible single-step procedure for the diagnosis of GDM [13].

The DIPSI method has been widely studied in the Indian scenario and was included in the guidelines issued by the Ministry of Health and Family Welfare, Government of India as a screening and diagnostic method for GDM [14]. Advantages of using DIPSI criteria in PW include the non-requirement for fasting that potentially may invoke morning sickness, fewer chances of nausea and/or vomiting after glucose load, and neither requires the PW to return for another test. This causes the least disturbance in PW's routine activities, can potentially diagnose pre-GDM, and serves as a preferred diagnostic procedure [15].

In the present study, which was conducted to compare the utility of DIPSI in comparison with the WHO criteria for GDM, the mean values of BMI were significantly higher in GDM patients compared to the non-GDM patients. An increase in BMI can result in insulin resistance, which contributes to increased blood glucose levels. This explains the glucose intolerance in patients with high BMI in the GDM group [16]. The mean values of second-hour blood glucose were significantly increased in GDM patients compared to non-GDM subjects. This may be attributed to insulin resistance and hyperglycemia consequent to high BMI, and other factors.

Out of the total 75 PW included in the study, 24 (32%) were diagnosed as suffering from GDM following the WHO and DIPSI guidelines. However, the WHO-based FBS estimation was able to diagnose 19 cases in contrast to the two cases identified by the second-hour blood glucose values after an OGTT. GDM was not diagnosed in any of the study participants after the first-hour cutoff values based on the WHO guidelines. The reason for higher FBS among the study population may be attributed to the higher BMI of the PW included for analysis.

Despite the efficacy of second-hour DIPSI values in identifying GDM, the low cutoff of 140 mg/dL compared to the 153 mg/dL for WHO criteria could still make it an inferior choice. In the present study, 19 of the 20 cases diagnosed by WHO criteria had increased FBS values. Of these 19 cases, only 11 were detected by the DIPSI criteria.

According to the HAPO study, higher isolated fasting glucose levels have a higher incidence of the occurrence of poor maternal and fetal outcomes such as fetal hyperglycemia, future diabetes, premature delivery, intensive neonatal care, hyperbilirubinemia, preeclampsia, shoulder dystocia, or birth injury [17].

Our study showed that the non-fasting DIPSI criteria may be inferior to the WHO guidelines in the diagnosis of GDM. There is an increasing chance of missed diagnosis when DIPSI criteria were applied alone. For a diagnostic test, missing such a large number is not acceptable since GDM is associated with both maternal and perinatal complications.

A study reported by Viswanathan Mohan et al., which included 1,031 PW attending antenatal outpatient department, inferred that DIPSI had poor sensitivity to diagnose GDM when compared to the WHO 1999 criteria and the IADPSG criteria. DIPSI criteria were found to miss more than 70% of PW with GDM who are otherwise diagnosed as suffering from GDM with the WHO criteria and the IADPSG criteria. This study concluded that the DIPSI non-fasting OGTT criteria cannot be recommended for the diagnosis of GDM [8].

In another Indian study reported from the state of Maharashtra, GDM was identified in only 6.5% of cases. It has been suggested that the lower prevalence rates reported might be influenced by the low sensitivity of DIPSI criteria [18]. Another similar study concluded that IADPSG criteria are better to screen for GDM in India [19].

A study by Sujoy et al. suggested that DIPSI criteria cannot be implemented as a screening test because more cases were diagnosed with IADPSG criteria than DIPSI criteria [20]. It was observed that more number of cases were diagnosed based on the FBS activities in contrast to the 2-hour blood glucose after an OGTT, a finding that was in concordance with our study results.

However, there are additional studies that prove the DIPSI method to be a convenient screening and diagnostic test for identifying GDM. These studies have observed that a cutoff value ≥7.8 mmol/L at a 2-hour interval after an OGTT is sufficient to diagnose GDM and positively influence pregnancy outcomes both in terms of the mother's as well child's health. Mixed results were noted by a few other studies which found that the DIPSI criteria were highly sensitive, specific, and have greater diagnostic accuracy compared with the WHO guidelines [21-23].

The situation in developing countries like India demands women travel long distances to attend antenatal clinics. Hence, it has been felt by many obstetricians and physicians that calling all PW to come in a fasting state would be a great challenge. This is the reason why the DIPSI method is still practiced in India.

Study limitations

The major limitations of this study include low sample size and the diagnostic efficacy including the sensitivity, specificity, positive predictive value, and negative predictive value of the two methods applied in this study was not performed.

## Conclusions

The DIPSI method was found to be inferior to the WHO criteria for the diagnosis of GDM. Results of this study have also demonstrated that more GDM cases could be identified using the FBS values that are estimated in the WHO strategy. Although the DIPSI criteria are convenient with less number of interventions required for the patients and are extremely helpful in a rural setting, it could possibly miss many cases of GDM. This could be responsible for the risk of diabetes in mothers and children born to such mothers in the future. The risks must be outweighed by benefits and therefore DIPSI criteria are not recommended to be used alone for the diagnosis of GDM.

## Appendices

Study questionnaire

**Table 4:** Proforma for data collection

S.No.	
Registration Number:	Date:
Name:	Age:
Husband/Guardian Name:	Obstetric Formula: G P L A D
Address:	
Marital Life (yrs):	LMP:
EDD:	Gestational Weeks:
SEDD:	Height: (cm)
Weight: (kg)	BMI:
B.P: (mm of Hg) :	
Past History of Any Major Illness:	
Past History of Diabetes/Hypertension/Hypothyroidism/GDM	
Family History of Diabetes in Parents/Siblings:	
History of Intake of Any Drugs:	
Habits: Intake of Alcohol, History of Smoking, Other Habits	
Any Abnormal Findings in the General examination:	

Obstetric history

**Table 5:** Obstetric history

Birth order	Home/institutional delivery	Type of delivery	Weight of baby	Any congenital anomalies/birth defects	History of GDM/ Preeclampsia
1.					
2.					
3.					
4.					
5.					

Format to record the measuring parameters

**Table 6:** Format to record the measuring parameters

Date	Parameter	Result
	BMI (kg/m^2)^	
	OGTT with DIPSI guidelines; Random plasma glucose (mg/dL); Second-hour plasma glucose (mg/dL)	
	OGTT with WHO guidelines; Fasting plasma glucose (mg/dL); First-hour plasma glucose (mg/dL); Second-hour plasma glucose (mg/dL)	
